# ASSOCIATION OF MENINGITIS AND PERICARDITIS IN INVASIVE PNEUMOCOCCAL DISEASE: A RARE CASE

**DOI:** 10.1590/1984-0462/;2019;37;1;00009

**Published:** 2018-08-30

**Authors:** Tiago Henrique de Souza, José Antônio Nadal, Carlos Eduardo Lopes, Roberto José Negrão Nogueira

**Affiliations:** a Universidade Estadual de Campinas, Campinas, SP, Brasil.

**Keywords:** Pericarditis, Meningitis, Pneumococcal infections, Infant, Pediatrics, Pericardite, Meningite, Infecções pneumocócicas, Lactente, Pediatria

## Abstract

**Objective:**

To report a rare case of a child with invasive pneumococcal disease that presented meningitis associated with pericarditis.

**Case description:**

This report describes the unfavorable clinical course of a previously healthy 6-months-old female infant who initially presented symptoms of fever and respiratory problems. A chest X-ray revealed an increased cardiac area with no radiographic changes in the lungs. After identifying a pericardial effusion, the patient experienced seizures and went into coma. Pneumonia was excluded as a possibility during the clinical investigation. However, *Streptococcus pneumoniae* was identified in the cerebrospinal fluid and blood cultures. An initial neurological examination showed that the patient was brain dead, which was then later confirmed according to protocol.

**Comments:**

Purulent pericarditis has become a rare complication of invasive pneumococcal disease since the advent of antibiotic therapy. Patients with extensive pneumonia are primarily predisposed and, even with early and adequate treatment, are prone to high mortality rates. The association of pneumococcal meningitis and pericarditis is uncommon, and therefore difficult to diagnose. As such, diagnostic suspicion must be high in order to institute early treatment and increase survival.

## RESUMO

**Objetivo:**

Relatar um caso raro de uma criança com meningite associada a pericardite na doença pneumocócica invasiva.

**Descrição do caso:**

Este relato descreve uma evolução clínica desfavorável de um lactente feminino de 6 meses de idade, previamente hígido, que apresentou inicialmente sintomas respiratórios e febre. A radiografia de tórax revelou um aumento da área cardíaca sem alterações radiográficas nos pulmões. Após a identificação do derrame pericárdico, o paciente apresentou convulsões e entrou em coma. Pneumonia foi descartada durante a investigação clínica. Contudo, foi identificado *Streptococcus pneumoniae* nas culturas de líquor e sangue. O exame neurológico inicial foi compatível com morte encefálica, posteriormente confirmada pelo protocolo.

**Comentários:**

A pericardite purulenta tornou-se uma complicação rara da doença pneumocócica invasiva desde o advento da terapia antibiótica. Pacientes com pneumonia extensa são primariamente predispostos e, mesmo com tratamento adequado e precoce, estão sujeitos a altas taxas de mortalidade. A associação de meningite pneumocócica e pericardite é incomum e, portanto, de difícil diagnóstico. Por isso, uma alta suspeição diagnóstica é necessária para instituir o tratamento precoce e aumentar a sobrevida.

## INTRODUCTION

*Streptococcus pneumoniae* predicates a variety of pediatric diseases, such as invasive pneumococcal disease, which includes bacteremic pneumonia, meningitis, and sepsis. Fatality rates are widely variable, ranging from 11% to 60%, with 90% of deaths occurring in low-income countries.^1^ ^,^ ^2^ In systemic bacterial infections, purulent pericardial effusion may develop rapidly; however, it has became a rare complication of invasive pneumococcal disease since the era of antibiotics.^3^ ^,^ ^4^ The mortality rate of purulent pericarditis (PP) is up to 30%, despite aggressive drainage and prolonged antibiotic therapy, and especially if the diagnosis and treatment are delayed.^5^ Hence, a high index of clinical suspicion for early diagnosis and treatment is essential for better outcomes.

In this paper, we report on a case of early pericardial effusion in invasive pneumococcal disease with a fulminant clinical course.

## CASE DESCRIPTION

**Figure 1:**
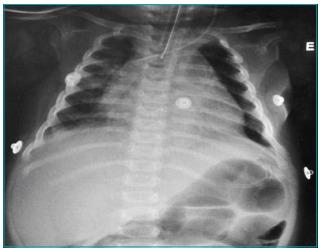
Chest x-ray.

A previously healthy 6-months-old female infant was referred to the Pediatric Intensive Care Unit of the Clinics Hospital of University of Campinas (CH-UNICAMP) because of a coma. Fifteen days prior to the coma, the patient had presented respiratory symptoms and had received antipyretics and short-acting B2-agonists. Four days prior to the coma, the patient presented respiratory distress and a fever, and was admitted to a secondary hospital in the metropolitan region of Campinas, São Paulo, Brazil. On the first day of hospitalization, the patient had a persistent fever and sinus tachycardia. A chest X-ray revealed an increased cardiac area with no radiographic changes in the lungs (Figure 1). The following day, an echocardiography revealed pericardial thickening with bulky effusion and signs of cardiac tamponade. The patient was reported to be in good general condition. On the third day of hospitalization, the patient suffered from seizures. Diazepam, phenobarbital, and phenytoin were administered, and an urgent transfer to the intensive care unit was requested. During transport, the patient was intubated and was administered dobutamine. Upon arrival to the CH-UNICAMP, the patient went into hypotensive shock, with a Glasgow coma score of 3.

**Figure 2:**
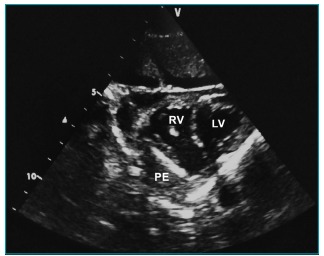
Cardiac ultrasound from a parasternal short axis view. Pericardial effusion with heterogeneous echogenicity and the presence of septations. LV: left ventricle; RV: right ventricle; PE: pericardial effusion.

After hemodynamic stabilization, a computed tomography (CT), a lumbar puncture, radiography, blood cultures, and laboratory tests were performed. The initial hemogram was: Hb 6.1 g/dL; Ht 19.8%; red blood cell count 3,320,000/mm^3^; white blood cell count 4290/mm^3^ (15.3% neutrophils; 12.2% band cells; 70.4% lymphocytes; 2.1% monocytes); platelet count 166000/mm^3^. A cerebrospinal fluid (CSF) analysis revealed: Proteins 1291 mg/dL; Blood cells 730/mm^3^; Glucose 0 ­mg/­dL; Leukocytes 51 cells/mm^3^ (86% neutrophils). Numerous gram-positive cocci were visualized in the CSF bacterioscopy. Ceftriaxone was administered to the patient at a dose of 100 mg/kg. For hemodynamic stabilization, epinephrine was required at an infusion rate of 0.3 mcg/kg/ min. The CT revealed diffuse cerebral edema and chest radiography showed a discrete peribronchial heterogeneous opacity in the right hemithorax and cardiomegaly. A bedside ultrasound showed a septate pericardial effusion (Figure 2) without pulmonary consolidation. The neurological examination showed that the patient was brain dead, and a pericardiocentesis was not performed. *Streptococcus pneumoniae* was identified in the blood and CSF cultures. The bacteria were susceptible to the following antibiotics: sulfamethoxazole-trimethoprim; benzylpenicillin; erythromycin; ceftriaxone; vancomycin; and levofloxacin. Brain death was confirmed according to protocol.^6^

## DISCUSSION

In the 1950s, over 50% of pericarditis cases were purulent. Currently, approximately 1-3% of pericarditis cases are purulent.^7^ ^,^ ^8^ The decrease can be attributed to the availability of antibiotic therapy, and particularly penicillin. Although *Streptococcus pneumoniae* is still one of the leading infectious species of pneumonia, meningitis, and sepsis in children worldwide, a small number of patients with pneumococcal pericarditis have been reported since 1995.^9^ Currently, the most common etiological agent of bacterial pericarditis is *Staphylococcus aureus*, but cases are also reported with *Haemophilus influenzae* and meningococcus infections.^10^

Purulent pericarditis is diagnosed when pus is drained from the pericardial space, or when bacteria are cultured from the pericardial fluid.^11^ The classic triad of symptoms, described by Claude Beck in 1935 (hypotension, jugular venous distention, and distant heart sounds) are found in up to 40% of all cases.^12^ ST segment elevation is a classic electrocardiographic feature of pericarditis, but is rarely found in purulent pericarditis.^10^ Most reported cases described either decreased voltage or no changes in the ST segment. Echocardiography and computed tomography may further aid in diagnosis. Echocardiography can be performed at the bedside, without the use of ionizing radiation, and at a low cost. Furthermore, echocardiography reveals multiple signs of cardiac tamponade and may identify the best site for pericardiocentesis. Although pericardial fluid analysis was not performed in the reported case, bands and loculations in the pericardial fluid were visualized using echocardiography. These ultrasound findings are suggestive of purulent pericarditis in the septic patient.^13^

The most common predisposing factor for purulent pericarditis is pneumonia, followed by osteomyelitis and septic arthritis.^10^ ^,^ ^14^ ^,^ ^15^ Pericarditis caused by pneumococci is often associated with pneumonia and empyema.^10^ ^,^ ^14^ Pathophysiology of these cases is still controversial. It is not clear whether the bacteria migrate into the pericardial sac directly from the intrathoracic infectious focus, or if the invasion occurs by hematogenous dissemination.^9^ ^,^ ^10^ ^,^ ^11^ ^,^ ^14^ ^,^ ^16^ The reported case presented initial symptoms compatible with pneumonia, but this was not evident on chest radiography. Subsequently, a chest ultrasound was performed, which ruled out the presence of pleural effusions or consolidations.^17^ We hypothesize that the initial symptoms of fever, cough, and wheezing may have resulted in myocardial involvement or a viral respiratory infection with subsequent pneumococcal invasion. The bacterial invasion of the pericardium likely occurred by hematogenous dissemination.

Major complications of purulent pericarditis are septicemia, cardiac tamponade, and constrictive pericarditis. Early antibiotic therapy and pericardial drainage are, therefore, essential. Antibiotics should be administered empirically until microbiological results are available. However, it is not always possible to cultivate the causative agent.^10^ ^,^ ^14^ In cases with loculated effusions, intrapericardial fibrinolytic therapy may be considered for adequate drainage before resorting to surgery.^8^ ^,^ ^10^ ^,^ ^14^ ^,^ ^18^ Pericardiotomy should be considered for dense adhesions, loculated or thick purulent effusion, tamponade recurrence, persistent infection, and progression to constriction.^8^ ^,^ ^18^ If not treated in time, purulent pericarditis has a mortality rate of nearly 100%.^19^ However, when medical and surgical treatments are combined, the mortality rate of purulent pericarditis is reduced to 20% or less.^20^

We found only one case reported in the literature of purulent pericarditis caused by pneumococcus in a 17 year-old girl with meningitis.^21^ The incidence of pericarditis in meningococcal disease (3-19%) is higher than that in invasive pneumococcal disease.^3^ ^,^ ^15^ Sagristà-Sauleda et al. identified 33 cases of purulent pericarditis in a hospital population of 593,601 in a 20-year period.^15^ Only one case occurred in a patient with meningitis, and it was identified from a post-mortem exam. There was no microbiological identification. No cases of purulent pericarditis were found in 896 German or 361 Indian pediatric patients with invasive pneumococcal disease.^4^ ^,^ ^22^

In conclusion, this report describes the unfavorable clinical course of a child who presented a fever and respiratory symptoms and was found to have pericarditis associated with meningitis. This association is uncommon and therefore difficult to diagnose. Thus, identifying signs of sepsis and prompt treatment is critical for better outcomes. It should also be emphasized that the clinical manifestations of bacterial meningitis in infants are nonspecific and this diagnosis should always be considered in febrile infants.

Despite its rarity since the advent of antibiotics, pediatric purulent pericarditis is a severe disease with a fulminant course. This diagnosis should always be considered when pericardial effusion is present in patients with infectious disease, especially pneumonia. The signs and symptoms vary and can be masked by other known comorbidities. A high index of diagnostic suspicion is necessary in order to institute early treatment and increase survival. Furthermore, patients with these symptoms should be reassigned to an intensive care setting for aggressive interventions and close monitoring.
